# Immunohistochemical investigation of canine lymph nodes collected during a rabies outbreak in South Africa

**DOI:** 10.1099/jgv.0.002166

**Published:** 2025-11-06

**Authors:** Redwan Rahmat, Matthijs F. Ravensberg, Debby Schipper, Keshia Kroh, Edwin J.B. Veldhuis Kroeze, Thijs Kuiken, Claude Sabeta, Corine H. GeurtsvanKessel, Carmen W.E. Embregts

**Affiliations:** 1 Department of Viroscience, Erasmus Medical Center, Rotterdam, Netherlands; 2 Veterinarian, Private sector, Port Elizabeth, South Africa; 3 Department of Veterinary Tropical Diseases, Faculty of Veterinary Science, University of Pretoria, Onderstepoort, Pretoria, South Africa

**Keywords:** B-cell response, immune depletion, lymph nodes, macrophage, rabies virus

## Abstract

Rabies is a fatal zoonosis that impairs host immune function, yet effects on peripheral lymphoid architecture are poorly defined. During a 2021–2022 rabies virus (RABV) outbreak in South Africa, we collected cervical lymph nodes from 36 rabies-suspect dogs; RABV RNA was detected in 27. Canine distemper virus RNA was detected in a subset across both RABV-positive (RABV+) and RABV-negative (RABV−) groups and was not associated with clinical-sign count. We set up a computer-assisted histological analysis tool to quantify germinal-centre (GC) nucleus density and immunohistochemistry for CD20, PNA and IBA1 to profile B cells, GC activity and macrophages. Within the outbreak cohort, GC density and marker-based metrics did not differ between RABV+ and RABV− dogs. Two healthy dogs were included as reference tissues; values in outbreak dogs were generally lower, but these contrasts are contextual given the limited, non-matched controls. This study provides a reproducible framework for quantifying immune cell organization in field-collected tissues during natural RABV exposure and highlights the need for larger, geographically matched control groups and complementary functional immune measurements.

**Impact Statement**

Rabies virus (RABV) is one of the most fatal yet understudied zoonotic viruses. While most research has focused on neuroinvasion and viral dissemination, much less is known about how natural rabies exposure affects immune tissues. This study shows the development of a computer-assisted histological analysis tool to investigate immune cell organization in dogs naturally exposed to RABV. It highlights the interpretive challenges associated with working with field-collected lymphoid tissues, particularly in stray animals with unknown vaccination status and variable health backgrounds. While no distinct immune depletion was found between RABV-positive and RABV-negative dogs, both groups exhibited a reduced number of stained immune cells compared to healthy controls. This study adds to a small but growing body of work examining how RABV interacts with the immune system outside the central nervous system.

## Introduction

Rabies is a zoonotic viral disease caused by species within the *Lyssavirus* genus of the *Rhabdoviridae* family [1]. While all currently known lyssaviruses can cause rabies in animals, only six are known to infect humans [2]. Rabies virus (RABV) poses the greatest public health risk, primarily due to its association with domestic dogs (*Canis lupus familiaris*). Domestic dogs are responsible for over 99% of human rabies cases, serving as the primary reservoir for RABV globally [3]. Although canine-transmitted rabies has been eradicated in certain regions, it remains a persistent threat worldwide [4].

Rabies causes an estimated 59,000 human deaths each year, with the majority of cases occurring in Africa and Asia, where vaccination coverage in domestic dog populations remains insufficient [5,6]. Despite the proven effectiveness of canine vaccination campaigns, socioeconomic and logistical barriers hinder sustained implementation in many low-resource settings [7,8]. These challenges were further compounded by the COVID-19 pandemic, which disrupted routine vaccination programmes and increased the risk of rabies outbreaks [9,12]. In South Africa, this was exemplified by the 2021 to 2022 outbreak in Nelson Mandela Bay Municipality, where low dog vaccination coverage led to hundreds of canine rabies cases and multiple human exposures [13]. In addition to RABV, several other infectious agents can cause canine encephalitis in South Africa, with canine distemper virus (CDV) commonly co-circulating in the same ecological settings [14,15]. CDV infection is characterized by neurological disease along with marked immunosuppression [16,17]. RABV, on the other hand, is classically described as immune-evasive via IFN-pathway antagonism by the P protein and limited activation of dendritic cells, which are associated with poor induction of virus-neutralizing antibodies (VNAs) [18,19]. Such muted priming could plausibly attenuate B-cell activation and germinal-centre (GC) responses.

RABV enters the host through the bite of an infected animal, initially infecting muscle and epithelial cells at the site of entry. However, this local replication is typically minimal and transient [20]. The virus quickly accesses peripheral nerves, where it undergoes retrograde axonal transport to the central nervous system (CNS), replicating extensively and causing fatal encephalitis [21]. Although RABV reaches high viral loads in the CNS, it is frequently associated with weak systemic immune responses, particularly early in disease, due to immune evasion strategies such as suppression of IFN signalling, inhibition of antigen presentation and modulation of cytokine responses. RABV rapidly enters peripheral nerves and ‘hides’ within neurons, thereby avoiding early immune detection and delaying antiviral responses [22]. This rapid neuronal entry contributes to the strikingly limited immune activation seen in early rabies infection, despite eventually high viral loads in the CNS [23,24]. Multiple immune evasion strategies, including suppression of IFN signalling, inhibition of antigen presentation and modulation of cytokine responses, further hinder both innate and adaptive immunity during disease progression [25,26].

While the pathological and immunosuppressive effects of RABV in the CNS are well described, including neuronal dysfunction and immune evasion, the dynamics of the systemic immune response, particularly within peripheral lymphoid organs, remain poorly understood. Experimental studies in dogs have shown that infection with wild-type RABV results in poor activation of dendritic and B cells and failure to generate VNAs, contributing to fatal outcomes [27]. Interestingly, not all RABV exposures result in lethal disease. Rabies-specific antibodies have occasionally been detected in unvaccinated, healthy domestic dogs, suggesting that non-lethal exposure or naturally acquired immunity may occur in rare instances [28]. These observations highlight the variability in immune responses among RABV-exposed dogs and may reflect differences in host susceptibility, viral strain or level and route of exposure.

Dogs are the principal reservoir for human rabies, yet the effects of RABV on peripheral lymph node architecture in naturally exposed dogs remain poorly defined. We hypothesized that RABV infection in naturally exposed dogs is associated with altered lymph node structure, reflected by changes in GC density, B-cell metrics, CD20-positive GC number and proportional area, PNA labelling of proliferating GC cells and macrophage distribution, consistent with systemic immunosuppression observed in experimental models [29,30]. To investigate this, we analysed cervical lymph nodes collected from dogs with suspected RABV infection during the 2021–2022 outbreak in Nelson Mandela Bay, South Africa, and classified animals as RABV-positive (RABV+) or RABV-negative (RABV−) by Reverse Transcription Quantitative Polymerase Chain Reaction (RT-qPCR on brain tissue. Two healthy dogs were included as reference tissues to provide baseline histology. We established a computer-assisted workflow combining immunohistochemistry for CD20 (B cells), PNA (GC activity) and IBA1 (macrophages) with quantitative digital image analysis [31,33]. GC density was quantified using a predefined grid-based sampling strategy as a proxy for lymphocyte content. Together, this approach allowed us to quantify changes in GC density, B-cell numbers and macrophage distribution, providing new insights into the impact of RABV on host immune function.

## Methods

### Animal material

Specimens were collected from 36 dogs in Nelson Mandela Bay, South Africa, that were euthanized after displaying clinical signs compatible with rabies, such as neurological symptoms. The cohort consisted of 22 males (61%) and 14 females (39%). Sterilization status was known for 19 dogs (18 unsterilized, 1 sterilized) and unknown for 17. Ownership status was unknown for 19 dogs; of the remaining 17, 15 were owned and 2 unowned. Vaccination records were available for only 3 dogs (1 vaccinated, 2 unvaccinated). Age was known for 33 animals, of which 30 were adults, 4 puppies and 2 of unknown age. Most dogs were in the medium weight class (10–20 kg, *n*=28), with smaller (*n*=5), larger (*n*=2) or unknown (*n*=1) weight classes also represented. Nutritional status was judged as good in 21 dogs and poor in 13, while unknown in 2. External signs such as bite marks were noted in 10 animals, and 14 dogs showed evidence of ectoparasites (mange, ticks or fleas). Housing conditions were reported as stray (*n*=18), enclosed (*n*=5), free-roaming (*n*=3) or unknown (*n*=10). An overview of the cohort is presented in Table S1, available in the online Supplementary Material.

Brain tissue (as sampled by the adapted straw method), parotid (salivary) gland and cervical lymph nodes were obtained at variable intervals post-euthanasia (ranging from 3 to 72 h). One part of each brain and parotid gland was preserved in RNAlater (QIAGEN), while the other parts and all lymph nodes were fixed in 10% neutral-buffered formalin for 14 days prior to processing for immunohistochemistry. In addition to the rabies-suspected dogs from South Africa, cervical lymph nodes were collected from two healthy control (HC) dogs that were euthanized under laboratory conditions in the Netherlands. These control dogs were not matched to the South African cohort in terms of geography or environment, and their tissues were included solely as reference samples to illustrate baseline lymph node architecture, not as a statistically powered comparator group. The tissues were fixed in 10% neutral-buffered formalin for 48 h post-euthanasia.

### RABV qPCR

An initial diagnostic PCR was performed in South Africa, followed by validation RT-qPCR testing at Erasmus Medical Center for the RABV+ brain and parotid gland samples. Briefly, RNA was isolated from the samples in RNAlater, using MiniRNA columns (QIAGEN) and including the on-column DNAse treatment. RT-qPCR using probes against RABV genotype-I was used to verify the presence of RABV RNA [34]. In parallel, SYBR Green-based qPCR targeting the nucleocapsid (N) gene was performed on brain tissue from all dogs to detect CDV [35].

### Immunohistochemistry

Immunohistochemical analysis was performed on 3 µm sections from formalin-fixed, paraffin-embedded brain, parotid gland and cervical lymph node tissues. Following standard deparaffinization and heat-induced antigen retrieval with citric acid (pH 6.0), sections were stained using the following primary antibodies: a mouse mAb against RABV nucleoprotein (RABV-N) (clone 5DF12) for brain and parotid gland sections [34] and mouse monoclonal anti-IBA1 (Thermo Fisher, MA5-27726), rabbit polyclonal anti-CD20 (Thermo Fisher, YA3773043) and biotinylated peanut agglutinin (PNA, Vector Laboratories, B1075-5) for cervical lymph node sections. HRP-conjugated secondary antibodies were used for IBA1 and CD20 detection, while a streptavidin-HRP conjugate was used for the PNA staining. Chromogen development was performed using AEC (3-amino-9-ethylcarbazole), and sections were counterstained with Mayer’s haematoxylin. Haematoxylin and Eosin (H and E) staining was additionally performed on all lymph node sections for routine histological assessment. Digital images were acquired using a Nanozoomer slide scanner system (Hamamatsu Photonics) with a 40× objective lens.

### Tissue analysis

Scanned brain and parotid gland sections stained for RABV-N were screened for the presence of RABV-N-positive staining. Digital slides of cervical lymph nodes were analysed using NDP.View2 software (Hamamatsu Photonics). For CD20 and PNA staining, GCs were manually annotated across the entire tissue section. GC distribution was determined by calculating the number of positively stained GCs per mm² of total lymph node area. In parallel, the percentage area of staining was calculated by dividing the total area of CD20- or PNA-positive GCs by the total lymph node area (see Fig. S1 for the annotation workflow). IBA1 expression was evaluated using a semi-quantitative scoring system ranging from 1 to 10, based on the distribution and extent of positively stained macrophage-rich regions across the lymphoid tissue (scoring rubric and examples in Fig. S2). Nucleus density analysis was performed on H- and E-stained cervical lymph node sections. Fifteen GCs per dog were randomly selected to ensure representative sampling and consistent tissue coverage. ImageJ software was used to quantify the number of nuclei within each selected GC using a standardized macro (grid selection and macro parameters in Fig. S3).

To ensure measurement consistency, all lymph node sections were evaluated for tissue preservation, with a focus on autolysis, which directly affects nuclear visualization. Sections were scored on a five-point scale from 1 (severely degraded) to 5 (well-preserved) based on structural integrity and degree of degradation (representative images and criteria in Fig. S4). Inflammatory infiltrates and hyperplasia were noted separately as indicators of immune activity or pathology but were not factored into the degradation score. Tissues assigned a score of 1 were excluded from all immunohistochemistry (IHC) analyses due to extensive degradation, including loss of architecture and post-mortem bacterial overgrowth. Sections with scores from 2 to 5 retained sufficient structural integrity to allow reliable interpretation. Following exclusion, a total of 22 RABV+ and 5 RABV− dogs were included in all analyses.

### Statistical analysis

Statistical analysis was performed for CD20 and PNA measurements (GC distribution and percentage of positively stained areas), the average nucleus density in the H- and E-stained cervical lymph node sections, as well as IBA1 immunohistochemistry scores. All comparisons were conducted using GraphPad Prism (GraphPad Software). As the data did not meet assumptions of normality, non-parametric testing was applied. For comparisons among three groups, the Kruskal–Wallis test with Dunn’s multiple comparisons was used. For unpaired two-group comparisons, the Mann–Whitney U test was used. Statistical significance was defined as *P*<0.05. For all other figures, data are presented descriptively as mean values with sd, without formal statistical comparison. Power analysis was performed using a two-sample t-test framework (two-sided, *α*=0.05, power=0.80). Power analysis details are provided in Table S2.

## Results

### Clinical signs, histopathology and molecular confirmation of rabies in clinical suspect dogs from South Africa

During the study period, 36 dogs with neurological signs suspected of rabies were included. Diagnostic PCR testing of brain and parotid gland tissues detected RABV RNA in 27 out of 36 dogs, while no RABV RNA was detected in the remaining 9 dogs. To explore alternative causes of neurological symptoms or potential co-infections, CDV qPCR was performed on brain tissue from all 36 dogs. CDV RNA was detected in 6 dogs, including 3 RABV+ and 3 RABV− animals. However, CDV detection did not show a clear pattern in relation to RABV status or clinical signs (Fig. S5). Clinical and demographic metadata such as nutritional status, weight and ectoparasite presence were assessed for potential associations with immune parameters. No association was observed between nutritional status and immune parameters (Fig. S6). While signs such as ataxia and paralysis were observed in CDV+ dogs, they also occurred frequently in RABV+ animals.

**Fig. 1.**
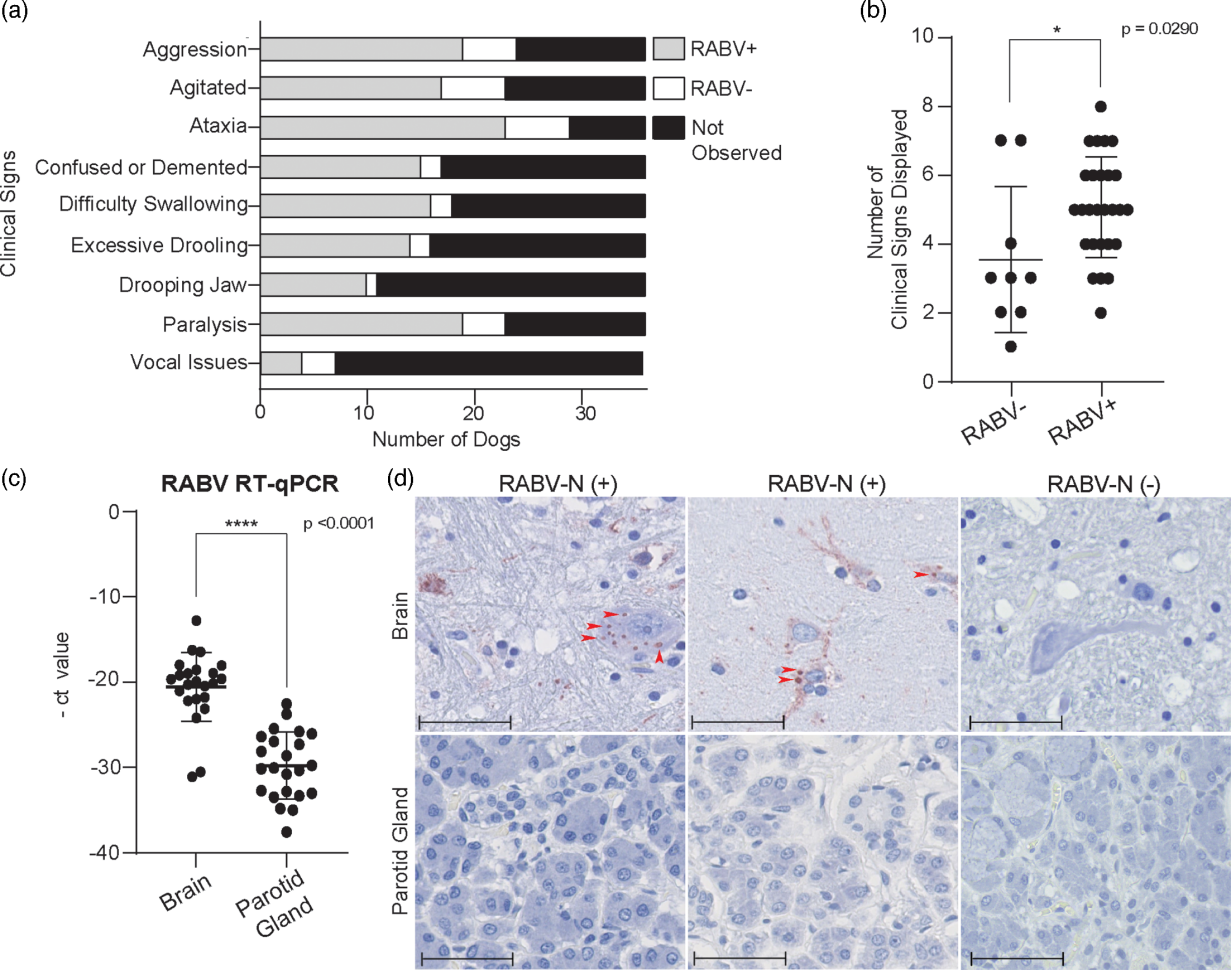
Clinical presentation, molecular confirmation and histopathological assessment of rabies in suspected dogs. (**a**) Frequency of observed clinical signs in RABV+ (*n*=27) and RABV− (*n*=9) dogs, including the ‘Not Observed’ category in black, which indicates the number of dogs in which the specified symptom was not observed. (**b**) Total number of clinical signs per dog, grouped by rabies status. (**c**) RT-qPCR analysis of RABV RNA in brain and parotid gland tissues, represented as -Ct values. All graphs display mean values, with error bars representing sd. (**d**) Representative immunohistochemistry for RABV-N in brain (top row) and parotid gland (bottom row). The two left columns show RABV+ dogs; the right column shows an RABV− dog. Arrowheads in the RABV+ dogs show neuronal inclusions. Scale bar, 50 µm.

Clinical signs associated with neurological progression and late-stage rabies were compared between RABV+ and RABV− dogs (Fig. 1a). In RABV+ dogs, observed signs were ataxia (85.2%), aggression (70.4%), paralysis (70.4%), agitation (63.0%), difficulty swallowing (59.3%), excessive drooling (51.9%), demented behaviour (55.6%), drooping jaw (37.0%) and vocal abnormalities (14.8%). In RABV− dogs, recorded signs were agitation (66.7%), ataxia (66.7%), aggression (55.6%), excessive drooling (44.4%), paralysis (44.4%), difficulty swallowing (22.2%), demented behaviour (22.2%), vocal abnormalities (33.3%) and drooping jaw (11.1%). Overall, RABV+ dogs displayed a greater number of clinical signs per animal (median 5, range: 2–8) than RABV− dogs (median 3, range: 1–7) (Fig. 1b).

Validation RT-qPCR revealed lower Ct values in brain samples (median 19.9, range: 12.8–31.1) than in the parotid gland (median 30.1, range: 22.6–37.6), indicating higher viral loads in the brain (Fig. 1c). RABV-N staining was detected throughout the brain tissue of all RABV+ dogs. Representative immunostaining images are shown from cerebrum sections (Fig. 1d). No RABV-N staining was detected in the parotid gland sections of any dog.

### GCs in RABV+ and RABV− dogs exhibit comparable nucleus densities

**Fig. 2.**
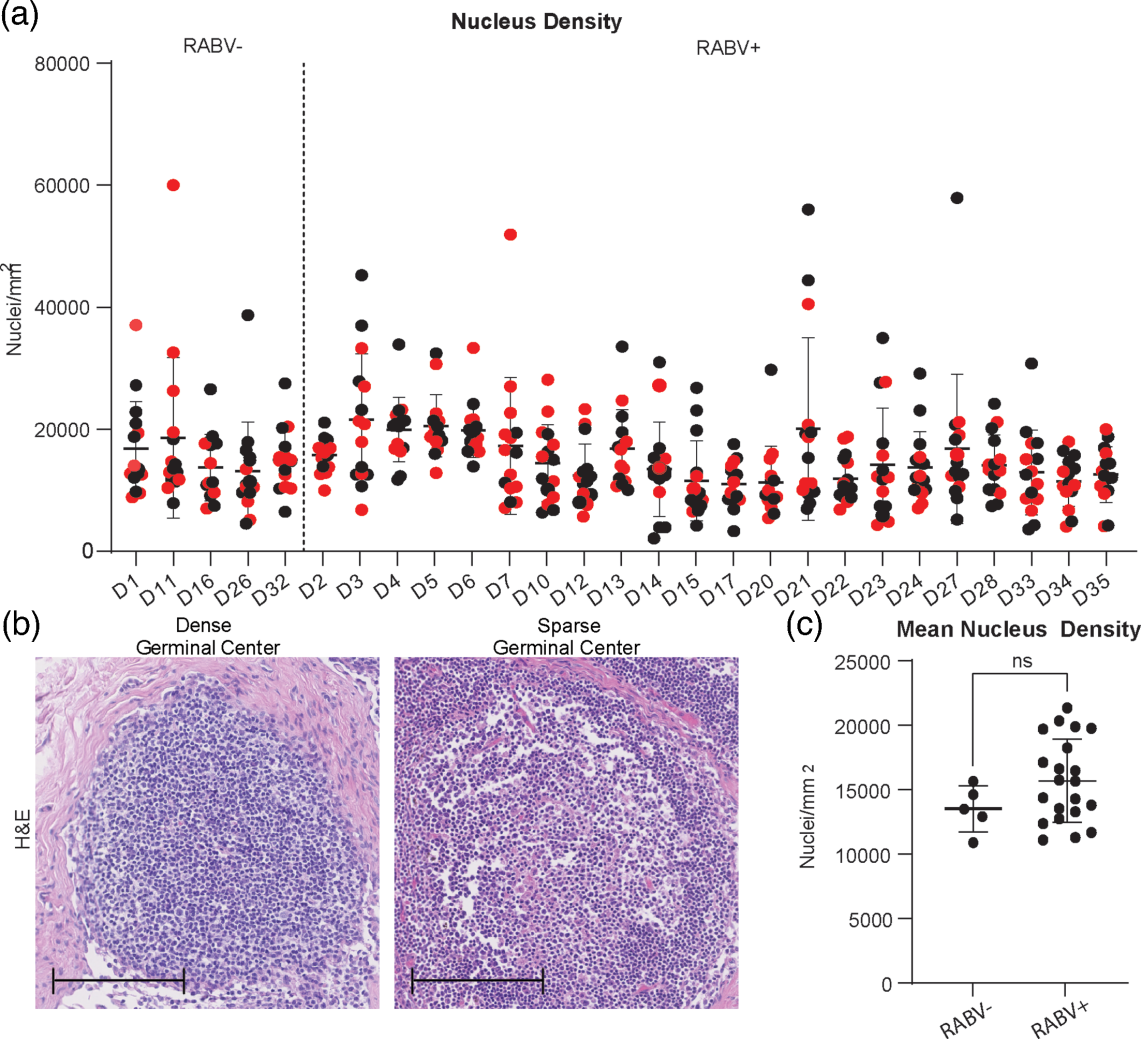
Nucleus density in GCs of cervical lymph nodes of RABV+ and RABV− dogs as assessed through H and E staining. (**a**) Distribution of nucleus densities across 15 GCs per lymph node. Red dots indicate primary follicles. (**b**) Representative images of GCs with dense and sparse nucleus distributions. Scale bar: 100 µm. (**c**) Mean nucleus density (nucleus mm^−^²) averaged across 15 GCs per lymph node in RABV− (*n*=5) and RABV+ (*n*=22) dogs. All graphs display mean values, with error bars representing sd.

Nucleus density was analysed in GCs of cervical lymph nodes using H- and E-stained sections. For each dog, 15 GCs were randomly selected using a grid-based approach (Fig. S3), and average nucleus counts per mm² were calculated as a proxy for lymphocyte density. Only lymph node sections with sufficient tissue integrity (score>1) were included for this and subsequent analysis, based on histological preservation and absence of overt degradation (Fig. S4),

To explore variation in GC nucleus density, individual GC densities across RABV+ and RABV− dogs are plotted in Fig. 2(a). Primary lymphoid follicles, identified based on their anatomical location at the outer edge of the lymph node cortex, did not display a consistent trend in nucleus density relative to non-primary follicles, and statistical comparisons did not reveal significant differences between these groups (data not shown). Across the cohort, GC nuclei densities varied widely, ranging from dense (1.9×10⁴ nuclei mm^−^²) to sparse (5.5×10³ nuclei mm^−^²) distributions (Fig. 2b), reflecting heterogeneity in GC architecture. Mean GC nucleus density per lymph node was not significantly different between RABV+ (1.6×10⁴±1.2×10³ nuclei mm^−^²) and RABV− dogs (1.5×10⁴±1.1×10³ nuclei mm^−^²) (Fig. 2c), indicating that RABV infection status alone did not drive measurable depletion. HC tissues exhibited higher mean nucleus density than outbreak dogs (Fig. S7). HC tissues are included as baseline references.

### Comparable GC responses in cervical lymph nodes of RABV+ and RABV− dogs

**Fig. 3.**
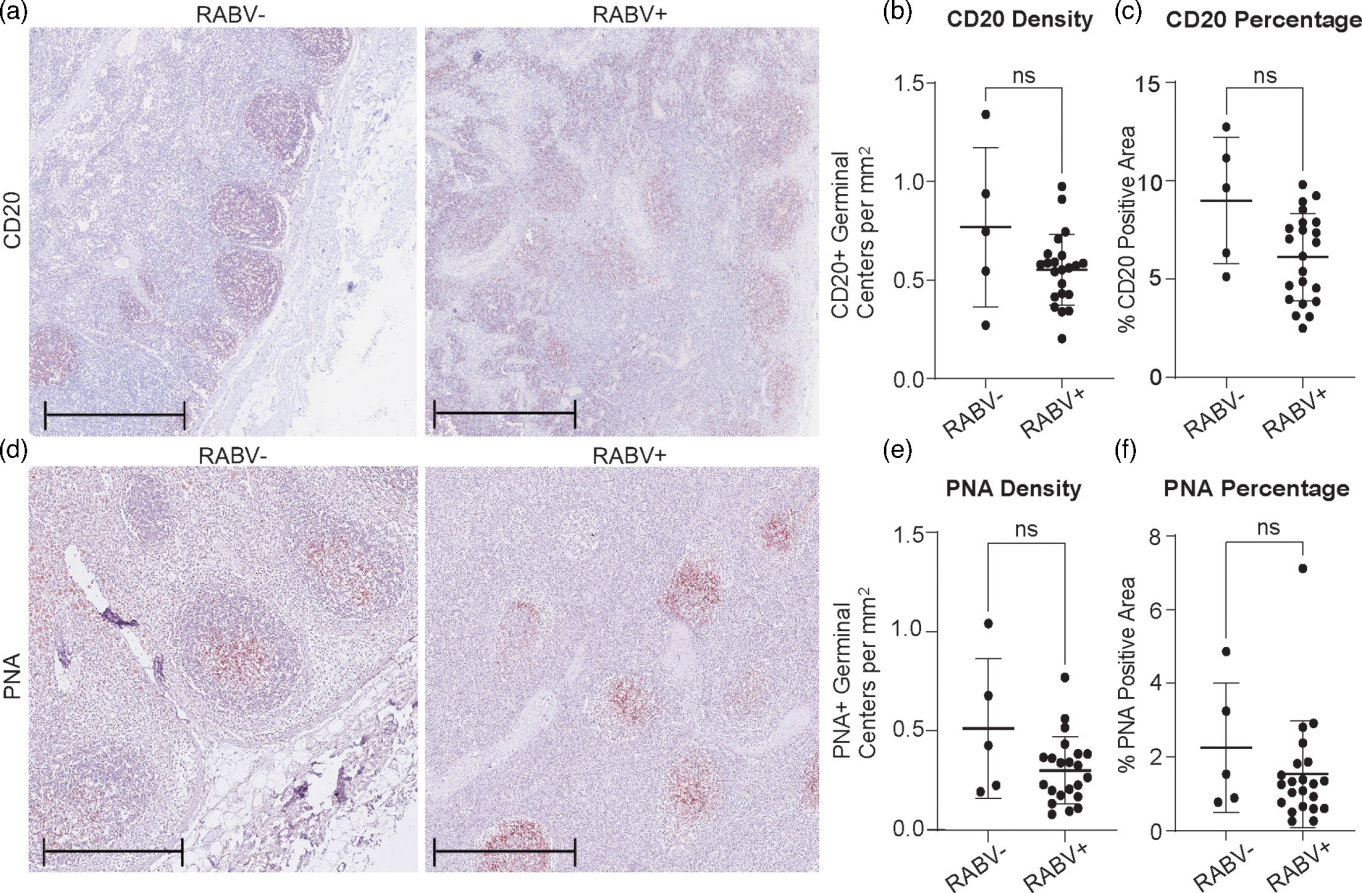
CD20 and PNA staining of cervical lymph nodes of RABV+ and RABV− dogs. (**a**) Representative images of CD20-stained (in red) cervical lymph nodes from RABV− and RABV+ dogs; scale bar: 500 µm. (**b**) Total number of CD20-positive GCs per mm² of lymph node tissue in RABV− dogs and RABV+. (**c**) Percentage of CD20-positive area relative to the total lymph node area in RABV− and RABV+ dogs. (**d**) Representative images of PNA-stained (in red) cervical lymph nodes from RABV− and RABV+ dogs; scale bar: 500 µm. (**e**) Total number of PNA-positive GCs per mm² of lymph node tissue in RABV− dogs and RABV+. (**f**) Percentage of PNA-positive area relative to the total lymph node area in RABV− dogs and RABV+. All graphs display mean values, with error bars sd.

Cervical lymph nodes were analysed to evaluate potential immune suppression in RABV+ and RABV− dogs, focusing on B-cell activity and GC proliferation. CD20 staining was used to quantify B cells in GCs (Fig. 3a). The number of CD20-positive GCs per mm² of lymph node tissue did not significantly differ between RABV+ (0.55±0.04 mm^−^²) and RABV− dogs (0.73±0.14 mm^−^²) (Fig. 3b). The percentage of CD20-positive GCs relative to the total lymph node area likewise showed comparable values between groups (RABV+: 6.33±0.29%; RABV−: 7.66±1.51%) (Fig. 3c). Proliferative activity was assessed using PNA staining (Fig. 3d). The number of PNA-positive GCs per mm² was 0.35±0.03 mm^−^² in RABV+ dogs and 0.53±0.15 mm^−^² in RABV− dogs (Fig. 3e). The percentage area of PNA-positive staining relative to total lymph node tissue was also similar between groups (RABV+: 1.84±0.20%; RABV−: 2.25±0.83%) (Fig. 3f). All comparisons were analysed using the Kruskal–Wallis test with Dunn’s multiple comparisons.

Within the outbreak cohort, CD20 and PNA metrics did not differ between RABV+ and RABV− dogs. Compared with HC reference tissues, which were from a different geographic region and were only used as reference values, animals from our South Africa cohort showed lower CD20-positive GC number and area, with PNA showing fewer GCs, while the percentage of PNA-positive area was comparable across groups (Fig. S8).

### No distinct differences in macrophage distribution between RABV+ and RABV− dogs

**Fig. 4.**
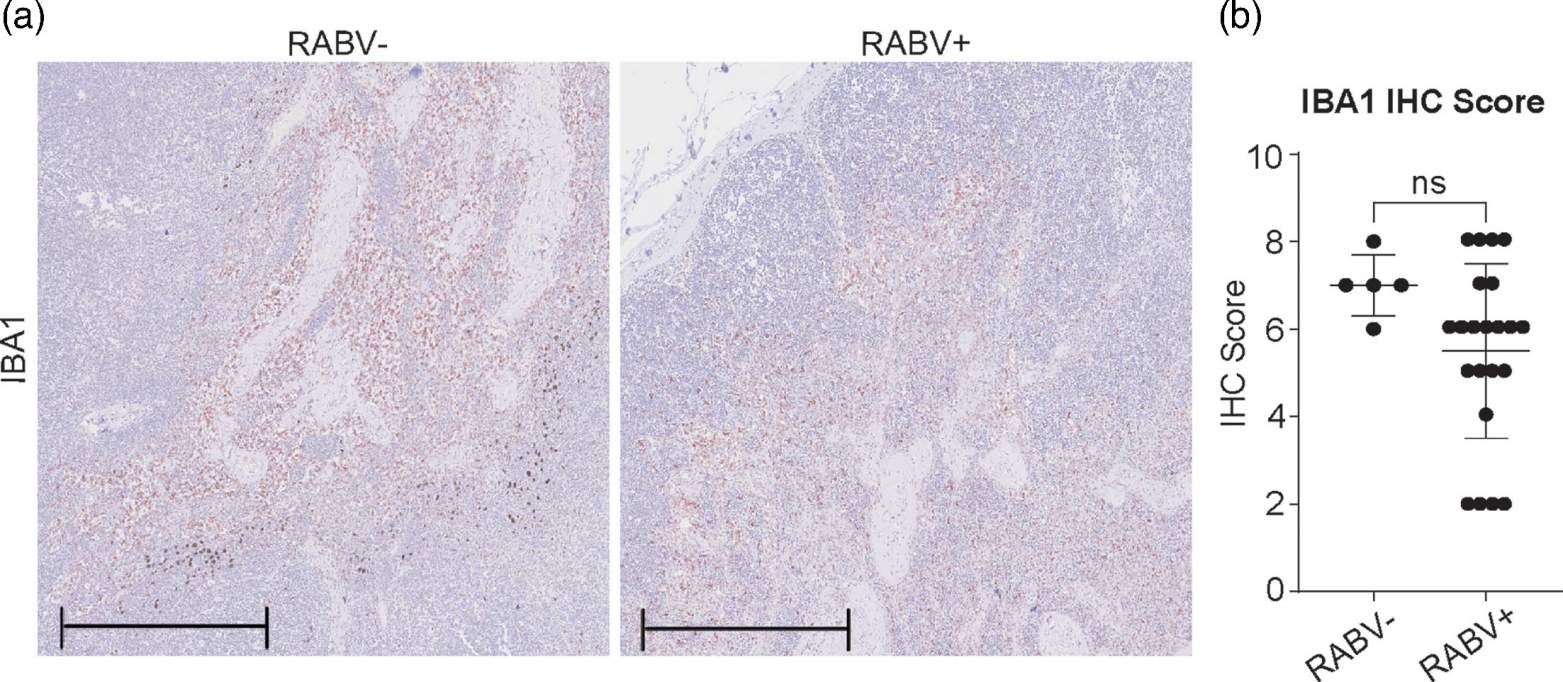
IBA1 staining of cervical lymph nodes in RABV+ and RABV− dogs. (**a**) Representative images of IBA1-stained (in red) cervical lymph nodes from RABV− and RABV+ dogs; scale bar: 500 µm (**b**) Histological scoring of IBA1 staining (1–10 scale) in RABV− dogs and RABV+. All graphs display mean values, with error bars representing sd.

To evaluate macrophage distribution in cervical lymph nodes, IBA1 IHC was performed (Fig. 4a). A semi-quantitative scoring system ranging from 1 to 10 was based on predefined criteria to assess the density and distribution of IBA1-positive cells across the tissue (Fig. S2). This scoring reflects the extent of macrophage presence across the lymph node tissue but does not measure activation or functional status. Among outbreak dogs, IBA1 scores ranged from 2 to 8 in RABV+ cases (median: 6) and from 6 to 8 in RABV− cases (median: 7), with no statistically significant difference between the two groups (Fig. 4b).

To provide baseline context, cervical lymph nodes from HC dogs were also examined (Fig. S9). In HC samples, IBA1-positive macrophages showed a broader and more uniform distribution, with staining evident throughout the cortex, paracortex and medullary cords. In contrast, both RABV+ and RABV− outbreak samples exhibited a more restricted IBA1 staining pattern, typically confined to interfollicular and medullary regions. Consistent with this difference, HC tissues received higher IBA1 histology scores than outbreak samples.

## Discussion

RABV is a neurotropic virus that causes fatal encephalitis. Upon infection, progression to the CNS occurs with minimal peripheral inflammation and a striking lack of antiviral immune responses. Most individuals infected with rabies, including humans and other mammals, fail to mount effective VNA responses [36], an outcome that has been consistently linked to poor prognosis and fatality. The inability to generate protective antibody responses highlights the critical role of effective B-cell activation and GC formation, processes that involve B-cell proliferation, somatic hypermutation, affinity maturation and class switching, in mounting a successful immune response [37]. Understanding how RABV modulates immune activity in these primary reservoirs during natural infections is critical to unravelling the broader host–pathogen dynamics. Yet, the immune responses of dogs naturally exposed to RABV have received limited attention.

In this study, we aimed to assess whether RABV infection in naturally exposed dogs leads to alterations in GC architecture and immune cell populations in cervical lymph nodes. To evaluate immune cell depletion, we implemented a grid-based selection strategy and performed quantitative analysis of GC nucleus density. This approach allowed us to explore whether differences in GC structure and cellular composition could explain the deficient antibody responses observed in most RABV− infected individuals and animals. We also assessed immune cell distribution using IHC markers for B cells (CD20), proliferating GC cells (PNA) and macrophages (IBA1) in RABV+ and RABV− dogs, with healthy dogs from the Netherlands serving as reference controls to establish baseline immune marker expression.

B cells play a key role in antiviral defence through the generation of high-affinity virus VNAs [32]. Since VNA production depends on effective GC formation, disrupted GC architecture has been proposed as a mechanism underlying the poor humoral responses frequently observed in RABV infections [27,38]. Experimental infections in mice have shown a systemic depletion of lymphocytes across multiple organs, including the thymus, spleen and lymph nodes, which was reversible in adrenalectomized animals, suggesting a role for neuroendocrine regulation in immune suppression [30]. This lymphoid depletion appeared to affect both B and T cell subsets and was associated with diminished cell proliferation and function [29,39]. Infected mice exhibited decreased counts of CD4+ and CD8+ T cells as well as B cells, particularly in lymph nodes and spleen. Given these observations in experimental models, we hypothesized that similar immune depletion might be detectable in naturally infected animals. To explore this possibility, we assessed GC nucleus density as a proxy for lymphocyte content but found no significant differences between RABV+ and RABV− dogs. We did not observe a distinct clustering by RABV status; rather, variability within groups appeared to outweigh any separation attributable to infection status. Nucleus densities in RABV− dogs appeared lower than in HC tissues, a pattern that we interpret cautiously, as it may reflect baseline differences in lymphoid cellularity or altered follicular organization characteristic of dogs sampled under outbreak conditions, rather than a direct effect of RABV infection [40]. CD20 and PNA measurements did not differ within the outbreak cohort. Apparent reductions relative to HC tissues are noted but treated as contextual observations in light of control-group constraints. While not included in the current study due to tissue limitations, direct analysis of GC-resident cell types, such as follicular helper T cells and follicular dendritic cells, may provide further insights into GC maintenance and antibody affinity maturation [41,42]. Importantly, the limited sample size in our study restricts the ability to conclusively confirm or refute our central hypothesis regarding RABV-associated immune depletion. Future studies with larger cohorts will be essential to more robustly test this hypothesis.

Macrophages are essential sentinels of the immune system, serving as first responders during viral infections. They initiate and shape the antiviral immune response through the production of cytokines, antigen presentation and recruitment of other immune cells [43]. Upon sensing pathogens, macrophages typically upregulate inflammatory mediators such as TNF-*α*, IL-6 and IL-12, which are critical for activating innate and adaptive immunity. In the context of rabies, RABV has been shown to modulate macrophage function and impair inflammatory signalling. Transcriptomic profiling has shown that RABV-exposed human macrophages develop a distinct activation phenotype, characterized by strong induction of antiviral and IFN-related genes. While some immune-dampening features such as IL-10 and TIFAB were also upregulated, the overall signature indicates activation rather than suppression [44]. Additionally, RABV can dampen pro-inflammatory cytokine release by engaging the cholinergic anti-inflammatory pathway through binding to nicotinic acetylcholine receptor *α*7 (nAChR*α*7), thereby reducing TNF-*α* and IL-6 production [45]. Macrophage scores did not differ by RABV status within the outbreak cohort. Qualitatively, lymph nodes from the South Africa dogs (both RABV+ and RABV−) showed macrophage staining concentrated in interfollicular and medullary regions, in contrast to the broader cortical and paracortical distribution typical of lymph nodes from HCs. This distributional pattern may indicate shifts in lymph node organization or immune activation associated with the outbreak context, and we view it as complementary to the quantitative scoring.

Our analysis focuses on structural and distributional markers and does not include functional immune readouts. Because only formalin-fixed tissues were available and no paired serum or viable cells were obtained from field-collected, post-mortem dogs, cytokine or chemokine profiling, VNA litres or antigen-specific cellular responses could not be assessed. Accordingly, the present findings should be interpreted as qualitative observations rather than direct evidence of immune depletion. Future studies that integrate paired serology and formalin-fixed paraffin-embedded (FFPE)-compatible approaches (multiplex immunofluorescence, RNA *in situ* hybridization or spatial transcriptomics) would better link structure with function.

Dogs in this study were mostly strays, with unknown vaccination status, exposure history and prior medical care. Such factors limit the ability to assess pre-existing infections, antigen exposure or illnesses. While most were assessed to be in good nutritional condition, no differences in measured immune parameters were observed across nutritional categories. To explore alternative causes of neurological symptoms in rabies-suspect animals, particularly those testing negative for RABV, a CDV qPCR was performed on brain tissue from all dogs. CDV RNA was detected in six animals, in both RABV− and RABV+ groups. Among RABV− dogs, this may explain the neurological presentation observed in some animals. Detection of CDV RNA in RABV+ dogs, however, is more difficult to interpret and raises the possibility of concurrent infection. While CDV is known to cause neurological signs and lymphoid depletion [16], no consistent pattern emerged in relation to clinical presentation in this cohort. To our knowledge, detection of RABV–CDV infection has not been definitively documented in domestic dogs. However, previous studies have shown that CDV can persist in the CNS, suggesting that its detection in these cases may reflect true viral persistence rather than incidental or residual RNA [46,47]. A subset of dogs displayed neurological symptoms despite testing negative for both RABV and CDV, leaving the aetiology unresolved. We did not perform a broader viral panel, as the study was designed to specifically assess CDV due to its potential immunosuppressive effects, which could bias interpretation of the immune measurements.

Interpretation relative to HCs is also limited by the small number of HC dogs and lack of geographic and environmental matching (Netherlands vs. South Africa). Access to healthy domestic dog lymph nodes is rare, as euthanasia of healthy animals is uncommon and experimental sampling is exceptional. In this study, HC tissues therefore served primarily as baseline histological references rather than a powered comparison. The most robust conclusion remains the absence of measurable differences between RABV+ and RABV− outbreak dogs; any contrasts with HCs should be regarded as descriptive. Tissue-related factors, including variability in preservation, prolonged fixation and differing post-euthanasia intervals, may have influenced antigen detection. Despite excluding samples of the poorest quality, residual variation remains a potential confounder. Additionally, the limited number of RABV− dogs constrained the statistical power to detect group differences, particularly for parameters with moderate effect sizes. Power estimates based on this cohort suggest that detecting statistically significant differences for most immune parameters would require 23 to 435 animals per group, depending on the marker analysed. This underscores the need for larger and more balanced comparison groups in future studies.

Altogether, our study does provide a systematic framework for analysing immune alterations in field-collected lymphoid tissues. By integrating IHC with computational quantification, we contribute to the understanding of how RABV may interact with host immunity in its primary reservoir species. Expanding such investigations may uncover new insights into rabies pathogenesis beyond its classical neurotropic profile and help clarify the factors that limit protective immune responses in naturally infected animals.

## Supplementary material

10.1099/jgv.0.002166 Uncited Supplementary Material 1.

## Abbreviations

CDV: canine distemper virus
CNS: central nervous system
GC: germinal centre
HC: healthy control
PNA: peanut agglutinin
RABV+: RABV-positive
RABV−: RABV-negative
RABV: rabies virus
VNAs: virus-neutralizing antibodies
