# Supplementary material for: Immunohistochemical investigation of canine lymph nodes collected during a rabies outbreak in South Africa
Source: J Gen Virol. 2025 Nov 6;106(11):002166. doi: 10.1099/jgv.0.002166 (PMC12591502; doi:10.1099/jgv.0.002166)
Supplement: Uncited Supplementary Material 1. [file jgv-106-02166-s001.pdf]

Supplementary

Variable	Category	Count (n)
Sex	Male	22
	Female	14
Sterilization	Yes	1
	No	18
	Unknown	17
Ownership	Owned	15
	Not owned	2
	Unknown	19
Vaccination	Vaccinated	1
	Not vaccinated	2
	Unknown	33
Bite Marks	Present	10
	None,	14
	Unknown	12
Age Group	Adults (≥1 year)	30
	Puppies (<1 year)	4
	Unknown	2
Weight Class	Large (>20 kg)	2
	Medium (10–20 kg)	28
	Small (<10 kg)	5
	Unknown	1
Nutritional Status	Good	21
	Poor	13
	Unknown	2
Housing Conditions	Enclosed	5
	Free-roaming	3
	Stray	18
	Unknown	10

Supplementary Table 1: Demographic and Clinical Characteristics of Rabies-Suspected Dog Specimens from South Africa (n = 36).

Parameter	Observed n (RABV+/ RABV-)	Cohen's d	Observed Power (1–β)	Required n per group for 80% power
**Nuclei Density**	22/5	0.56	0.19	51
**CD20 Density**	22/5	0.28	0.08	197
**CD20 Percentage**	22/5	1.2	0.64	12
**PNA Density**	22/5	0.19	0.07	435
**PNA Percentage**	22/5	0.48	0.15	68
**IBA1 IHC Score**	22/5	0.84	0.37	23

Supplementary Table 2: Post hoc power analysis for immune parameters comparing RABV+ and RABV− dogs. Effect sizes were calculated using Cohen's d; using the formula:

$$Cohen's\ d = \frac{[(Mean\ RABV +) - (Mean\ RABV -)]}{Pooled\ SD}$$

Power calculations were conducted using the TTestIndPower function from the statsmodels library in Python.

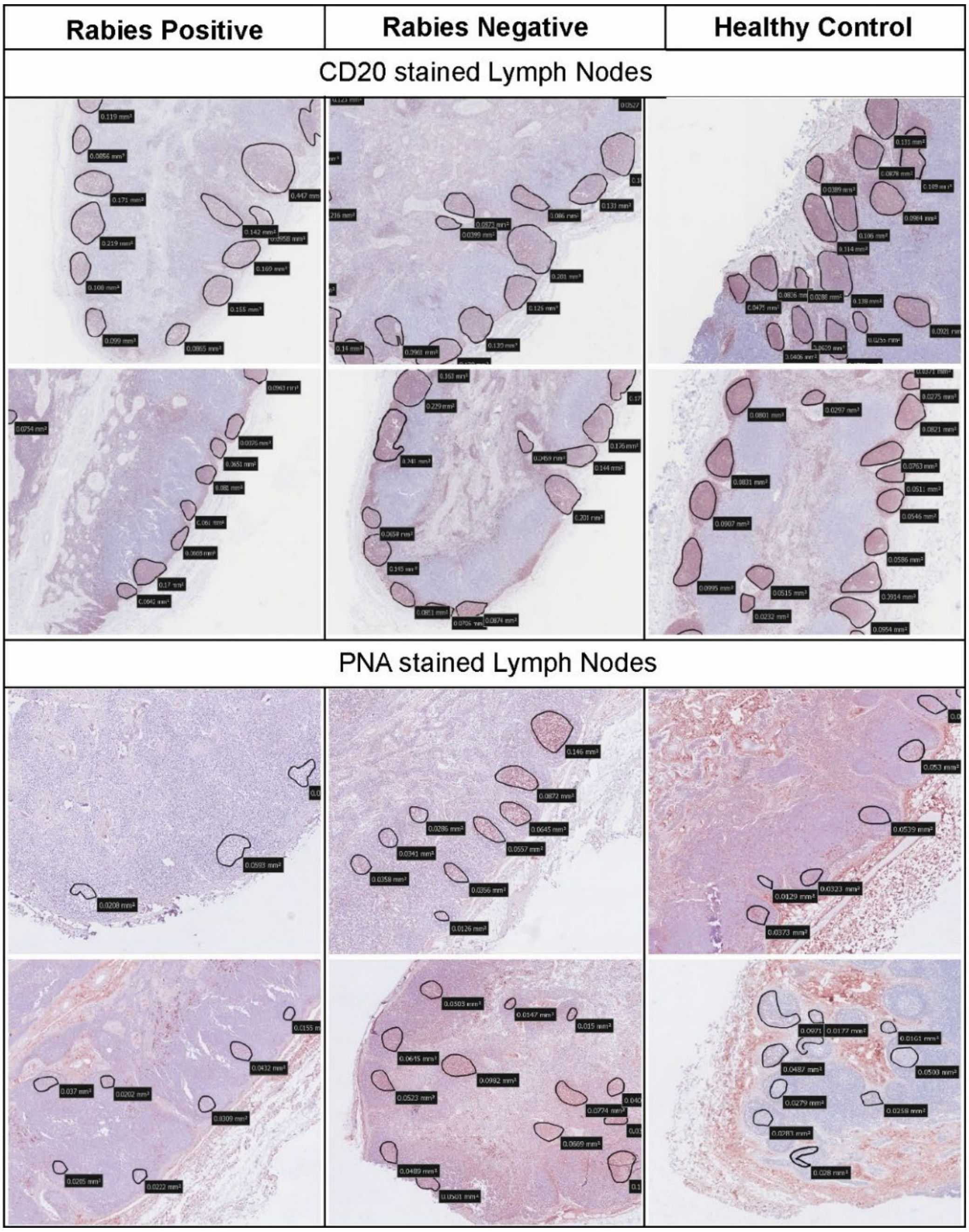

Supplementary Figure 1: Annotation and Measurement of GC Areas in CD20 and PNA-Stained Lymph Nodes. Methodology used to quantify the areas of GC areas in CD20- and PNA-stained lymph nodes.

Tissue Image	Remark	Assigned Score
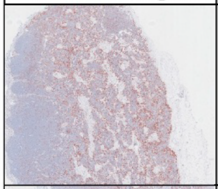	Intense and uniform IBA1 staining throughout the tissue section.<br>Indicates robust macrophage presence with no variability in staining intensity.	10
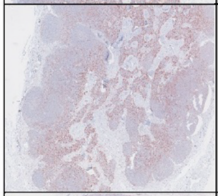	Strong IBA1 staining across the tissue, with very minimal variability.<br>Slight reduction in staining uniformity compared to Score 10	9
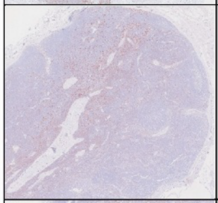	Strong IBA1 staining with slight variations in intensity across different regions.<br>Maintains overall good macrophage distribution.	8
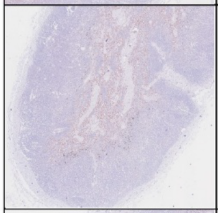	Noticeable reductions in IBA1 staining intensity in some regions, with overall macrophage presence still evident.	7
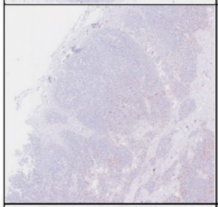	Moderate IBA1 staining intensity with variability across regions.<br>Macrophage distribution remains observable but less consistent.	6
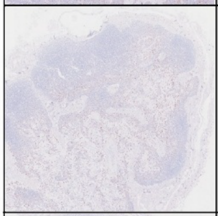	Moderate IBA1 presence, with noticeable variations in staining intensity and more irregular distribution patterns.	5
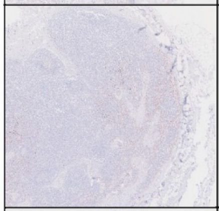	Reduced IBA1 staining intensity, with irregular distribution and areas showing minimal staining.	4
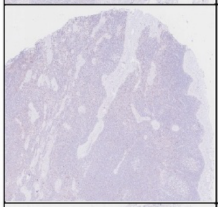	Low IBA1 intensity with patchy distribution patterns.<br>Indicates minimal macrophage presence in some areas.	3
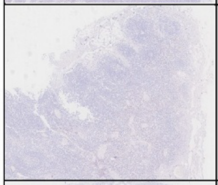	Minimal IBA1 staining observed, with large areas lacking positive staining.<br>Indicates severely reduced macrophage presence.	2
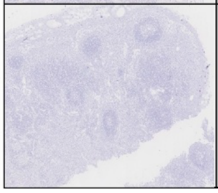	Almost no detectable IBA1 staining across the tissue section.<br>Indicates an absence or near absence of macrophages.	1

Supplementary Figure 2: Semi-Quantitative Scoring System for IBA1 Staining Intensity in Cervical Lymph Nodes. Standardized scoring system, developed to evaluate IBA1 staining intensity and distribution in cervical lymph nodes

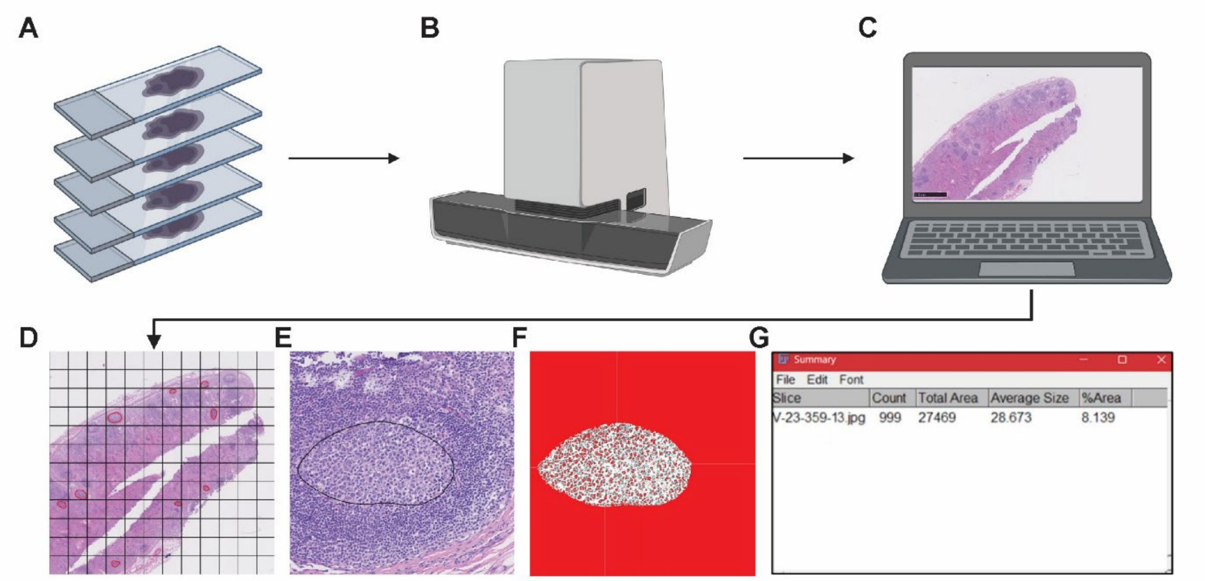

Supplementary Figure 3: Grid-based method used for random selection of GCs in lymph node sections (A) Batch of slides containing Lymph Node Tissues. (B) Tissues are scanned using Nanozoomer slide scanner system (Hamamatsu Photonics). (C) Tissues are visualized using NDP.View2 software (Hamamatsu Photonics). (D) A grid is created and germinal centers are randomly selected, shown annotated in red. (E) A close-up of one germinal center is shown. (F) The corresponding nuclei segmentation performed using a macro in Fiji ImageJ. (G) Count result as shown on Fiji ImageJ. Workflow created in https://BioRender.com

Tissue Image	Remark	Assigned Score
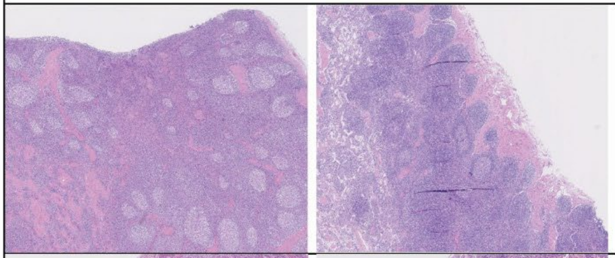	Lymph nodes with normal architecture, showing no signs of inflammation or other pathological changes	5
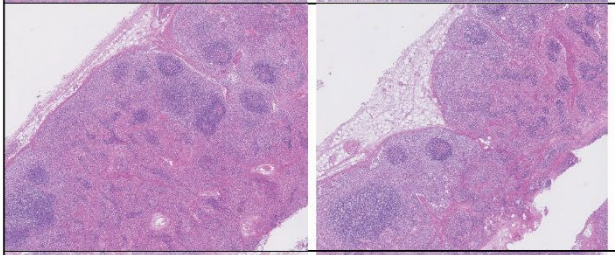	Lymph nodes that were slightly enlarged, with mild signs of inflammation but no significant structural alterations	4
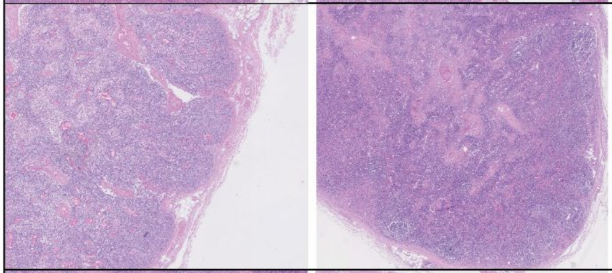	Hyperplastic lymph nodes, characterized by germinal center enlargement and activation	3
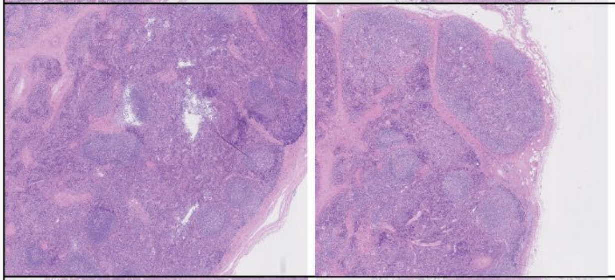	Hyperplastic lymph nodes with artefacts, including structural damage or poor preservation quality	2
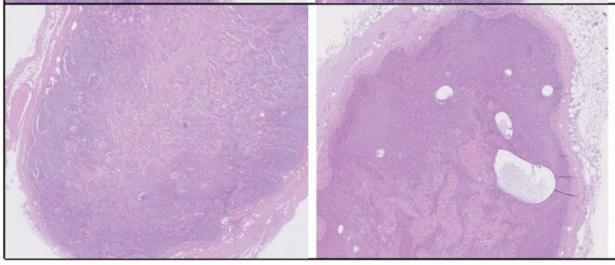	Severely degraded lymph nodes, displaying autolytic changes or post-mortem bacterial overgrowth, with substantial loss of cellular structure	1

Supplementary Figure 4: Histopathological Quality Assessment of Lymph Nodes. Lymph nodes from RABV+ and RABV− dogs were evaluated for tissue quality based on histopathological examination. Samples were scored on a scale of 1 to 5.

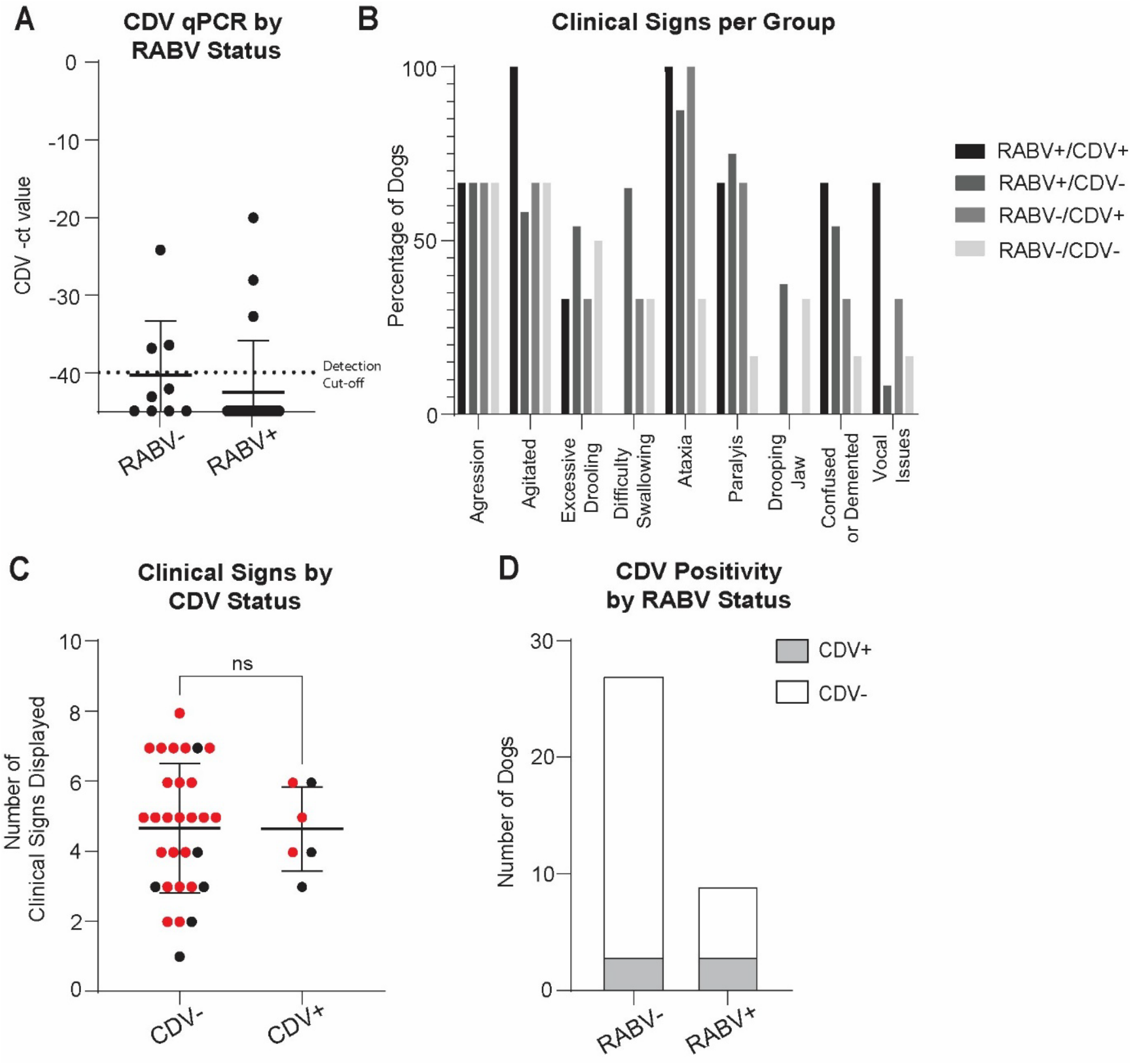

Supplementary Figure 5: CDV RT-qPCR Results in Rabies-Suspected Dogs. (A) CDV qPCR Ct values in CDV+ dogs, grouped by RABV+ (n=27) and RABV− (n=9). Lower Ct values correspond to higher viral RNA levels. (B) Percentage of dogs within each infection group (RABV+/CDV+, RABV+/CDV−, RABV−/CDV+, RABV−/CDV−) displaying each clinical sign. Group sizes were: RABV+/CDV+ (n=3), RABV+/CDV− (n=24), RABV−/CDV+ (n=3), and RABV−/CDV− (n=6). Percentages reflect the proportion of dogs per group showing a given sign. (C) Total number of clinical signs observed in CDV+ versus CDV− dogs. Red points represent RABV+, and black points represent RABV− dogs. Graphs display mean values, with error bars representing SD. (D) Number of CDV+ and CDV− dogs shown by RABV status

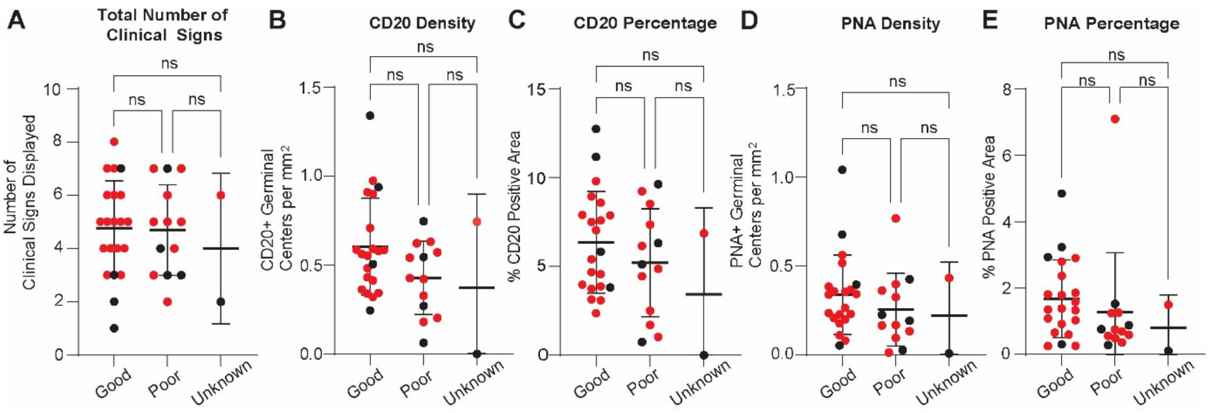

Supplementary Figure 6: Nutritional Status and Immune Marker Expression in Rabies-Suspected Dogs. (A) Total number of clinical signs observed per dog, grouped by nutritional status (good, n = 21; poor, n = 13; unknown. n=2). (B) Number of CD20-positive germinal centers per mm^2 of lymph node tissue. (C) Percentage of lymph node area occupied by CD20-positive germinal centers. (D) Number of PNA-positive germinal centers per mm^2 of lymph node tissue. (E) Percentage of lymph node area occupied by PNA-positive germinal centers. Red points represent RABV+, and black points represent RABV− dogs. All graphs display mean values, with error bars representing SD.

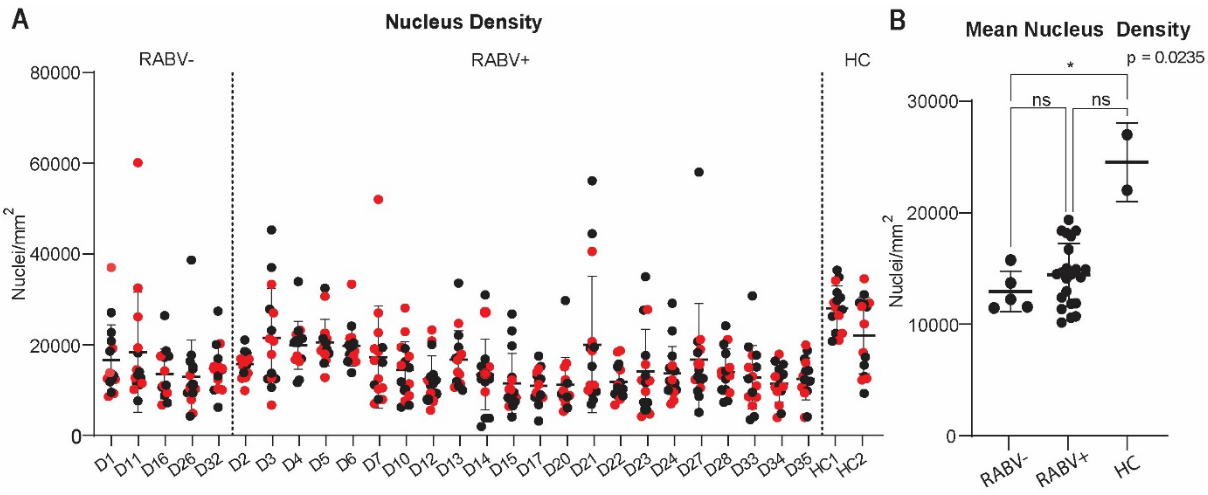

Supplementary Figure 7. GC Nuclei Density in Cervical Lymph Nodes of RABV+, RABV-, and HC Dogs. (A) Nuclei density in individual germinal centers from cervical lymph nodes of RABV−, RABV+, and HC dogs are shown. Each point represents one germinal center; RABV− and RABV+ dogs are separated by dashed lines on the left, and HC dogs (n = 2) appear on the right. Red dots indicate primary follicles. (B) Average nuclei density per tissue is summarized, where RABV+ (n = 22) and RABV− (n = 5) dogs showed similar values, while HC dogs exhibited higher densities, compared to RABV−. All graphs display mean values, with error bars representing SD.

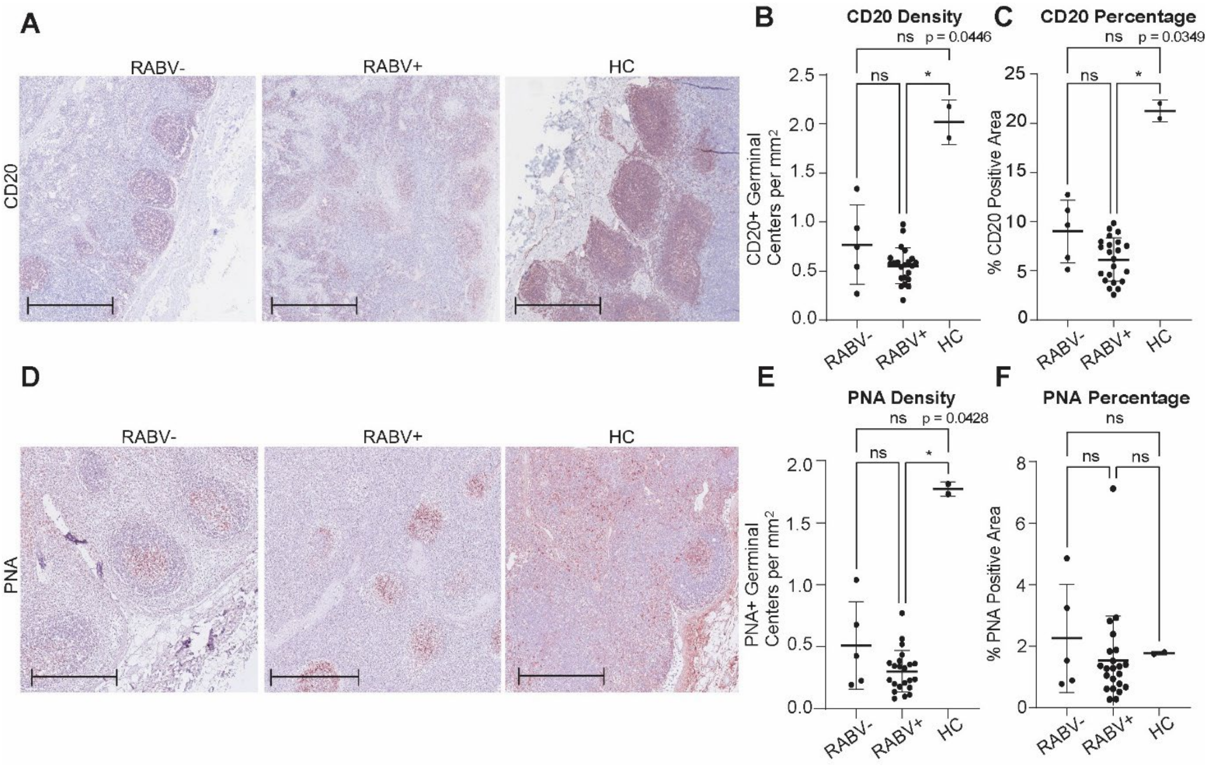

Supplementary Figure 8: CD20 and PNA Staining in Lymph Nodes of RABV+, RABV-, and HC Dogs. CD20 and PNA staining in cervical lymph nodes from RABV+ (n = 22), RABV− (n = 5), and HC (n = 2) dogs. (A) Representative CD20 staining in lymph nodes from each group. (B) The number of CD20-positive germinal centers per mm^2 (C) The percentage of CD20-positive area relative to total lymph node area. (D) Representative images of PNA staining across the three groups (E) The number of PNA-positive germinal centers per mm^2 (F) The percentage of PNA-positive area. Scale bars: 500 μm. All graphs display mean values, with error bars representing SD.

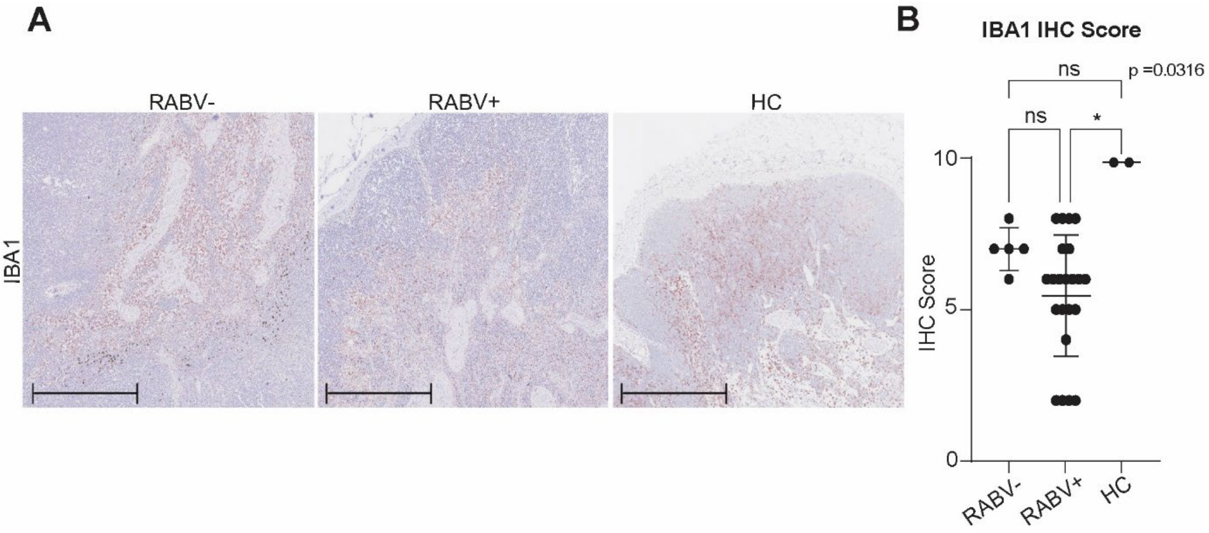

Supplementary Figure 9: IBA1 staining and semi-quantitative analysis of macrophage distribution in cervical lymph nodes from RABV−, RABV+ and HC dogs. IBA 1 staining in cervical lymph nodes from RABV+ (n = 22), RABV− (n = 5), and HC (n = 2) dogs. (A) Representative IBA1-stained lymph node sections from each group. (B) The semi-quantitative IHC scores assigned based on IBA1 staining intensity and distribution patterns. Scale bar: 500 µm. The graph displays mean values, with error bars representing SD.
